# A Case of Late-Onset Systemic Lupus Erythematosus With Systemic Symptoms Leading to Multiple Organ Failure

**DOI:** 10.7759/cureus.46428

**Published:** 2023-10-03

**Authors:** Tomoya Hasegawa, Kasumi Nishikawa, Yutaka Ohjino, Chiaki Sano, Ryuichi Ohta

**Affiliations:** 1 Cardiology, Matsue Seikyo General Hospital, Matsue, JPN; 2 Family Medicine, Shimane University Hospital, Izumo, JPN; 3 Family Medicine, Yonemori Hospital, Kagoshima, JPN; 4 Community Medicine Management, Shimane University Faculty of Medicine, Izumo, JPN; 5 Communiy Care, Unnan City Hospital, Unnan, JPN

**Keywords:** therapeutic intervention, organ dysfunction, comorbidities, multidisciplinary collaboration, immune-mediated vasculitis, diagnostic challenges, elderly patients, autoimmune disease, late-onset sle, systemic lupus erythematosus (sle)

## Abstract

This case report discusses the diagnosis and management of late-onset systemic lupus erythematosus (SLE) in an elderly patient. Systemic lupus erythematosus is an autoimmune disease that affects several organs. Sex differences in incidence, especially among women in their childbearing years, have been linked to estrogen fluctuations. This study focuses on an 87-year-old male who initially presented with anorexia, a history of heart failure, pancytopenia, and elevated antinuclear antibodies. His symptoms were initially attributed to heart failure and pneumonia. However, further evaluation led to the suspicion of immune-mediated vasculitis. Treatment with prednisolone improved his condition; however, a recurrent decrease in food intake and increased inflammation prompted the consideration of late-onset SLE. The diagnosis was supported by laboratory results, including antinuclear antibodies and complement levels, in accordance with the diagnostic criteria. This case highlights the challenges in diagnosing late-onset SLE owing to its overlap with other conditions and emphasizes the importance of a multidisciplinary approach for accurate diagnosis and treatment. Early recognition and intervention are crucial for managing late-onset SLE, even in elderly patients, to prevent multiple organ failure and improve outcomes.

## Introduction

Systemic lupus erythematosus (SLE) is primarily caused by acquired immunological abnormalities resulting from autoimmunity that affect tissues and organs throughout the body [1]. It causes inflammation in various organs, including the skin, joints, and kidneys [2]. Systemic lupus erythematosus is prevalent in the 20-40 year age group and is diagnosed 10 times more often in women [2]. Striking sex differences have been suggested to be related to estrogen levels, specifically the increase in SLE incidence during childbearing years, exacerbation during pregnancy, and resolution of symptoms after menopause, suggesting an influence of estrogen fluctuations on disease onset and progression [3]. Furthermore, age-related sex differences in SLE onset are intriguing. Women were more likely to develop the disease at a younger age, whereas men were more likely to develop it at both younger and older ages [4]. Multiple factors, including hormonal, genetic, and environmental, play complex roles in these sex differences [5]. Systemic lupus erythematosus is difficult to diagnose because it requires knowledge of a wide variety of symptoms and the recall of many differential diagnoses. Therefore, an accurate understanding of pathogenesis, a multifaceted approach, and a comprehensive perspective are essential.

Late-onset SLE is defined as SLE occurring at or above 50 years of age. The onset of SLE in the elderly is generally uncommon and often leads to a lengthy diagnostic process [6]. In many cases, diagnosis may be delayed because of the complexity of the disease and the many overlaps and similarities of its symptoms with those of other diseases [7]. However, once a diagnosis of late-onset SLE is confirmed, the response to treatment is generally good, with relatively minor organ damage reported [8]. Moreover, individual differences exist in the course of the disease, and the risk of severe disease in some patients due to repeated inflammation cannot be ignored [9]. In our case, an elderly patient presented with symptoms of multiple organ dysfunction syndrome on several occasions. After close examination and observation, a diagnosis of late-onset SLE was made. Although many diseases were considered at the initial diagnostic stage due to the variety of symptoms, ruling out each disease individually narrowed the diagnosis to late-onset SLE [9]. This case demonstrates that SLE diagnosis in elderly patients requires careful evaluation, continuous observation, and multidisciplinary collaboration. In this case study, we discuss approaches for diagnosing and managing systemic inflammatory diseases, particularly SLE, in the elderly population.

## Case presentation

An 87-year-old male who had been living at home and was independent in his daily activities visited our hospital complaining of anorexia. Three months prior, he visited our hospital with dyspnea and was diagnosed with congestive heart failure. The patient was discharged on the 14th day of hospitalization after symptom improvement following oral furosemide at 40 mg daily for one week. During his hospitalization, he had pancytopenia, his antinuclear antibody (nucleus) was 80-fold, and immunoglobin G was 2,077 mg/dL; therefore, he was followed up for suspicion of autoimmune hepatitis. Tests for antiribonucleoprotein, anti-Sm, and anti-double-stranded deoxyribonucleic acid antibodies yielded negative results.

One month before his visit, he was admitted to our hospital complaining of anorexia and was diagnosed with bacterial pneumonia. He was treated with ceftriaxone for pneumonia; however, his fever persisted and his anorexia did not improve. After thoroughly examining other potential causes of his symptoms, we suspected immune-mediated vasculitis. We initiated treatment with 50 mg of prednisolone due to low complement levels and increased urinary protein (C3: 85 mg/dL, C4: 13 mg/dL, estimated daily urinary protein: 15.1 g/1.73 m2). His fever resolved, and he could eat well; therefore, the prednisolone dose was reduced to 25 mg, and he was discharged on the 13th day of hospitalization. The prednisolone dose was subsequently reduced to 10 mg; however, the patient returned to our hospital one week later because his food intake had decreased. His medical history included a right-sided cardiogenic cerebral embolism, atrial fibrillation, gastrectomy for gastric ulcers, chronic kidney disease, and ascending aortic aneurysm. Medications included edoxaban (30 mg/day), silodosin (4 mg/day), empagliflozin (10 mg/day), spironolactone (25 mg/day), and prednisolone (10 mg/day).

On arrival at the hospital, vital signs were as follows: consciousness was slightly somnolent, the temperature was 36.3°C, blood pressure 137/107 mmHg, respiratory rate 22 breaths/min, pulse 101/min irregular, and oxygen saturation (SpO2) of 95% (room air). Physical examination revealed no neck rigidity; however, the eyelid conjunctiva appeared mildly pale, and the jugular vein was distended. Respiratory sounds were diminished on the right dorsal side; however, there were no wheezes or heart murmurs. The abdomen was flat and soft, with tenderness in the right hypochondrium. No leg edema, skin rash, or joint swelling were observed; however, peripheral coldness was observed. Blood tests revealed hepatic and renal dysfunction and a marked increase in brain natriuretic peptide levels (Table 1).

**Table 1:** Initial laboratory tests eGFR: estimated glomerular filtration rate; Na: sodium; K: potassium; Cl: chloride; Ca: calcium; P: phosphorus; Mg: magnesium; CK: creatine kinase; CK-MB: creatine kinase-myoglobin binding; TSH, thyroid-stimulating hormone; TSH: thyroid-stimulating hormone; T4: thyroxine

Parameters	Level	Reference range
White blood cells	10.30	3.5–9.1 × 10^3^/μL
Neutrophils	93.2	44.0–72.0%
Lymphocytes	1.5	18.0–59.0%
Monocytes	3.6	0.0–12.0%
Eosinophils	0.0	0.0–10.0%
Basophils	1.7	0.0–3.0%
Red blood cells	4.45	3.76–5.50 × 10^6^/μL
Hemoglobin	14.1	11.3–15.2 g/dL
Hematocrit	42.7	33.4–44.9%
Mean corpuscular volume	95.9	79.0–100.0 fL
Platelets	14.4	13.0–36.9 × 10^4^/μL
Total protein	6.4	6.5–8.3 g/dL
Albumin	3.0	3.8–5.3 g/dL
Total bilirubin	2.5	0.2–1.2 mg/dL
Direct bilirubin	1.7	0.0–0.4 mg/dL
Aspartate aminotransferase	424	8–38 IU/L
Alanine aminotransferase	430	4–43 IU/L
Alkaline phosphatase	141	106–322 U/L
γ-Glutamyl transpeptidase	62	<48 IU/L
Lactate dehydrogenase	645	121–245 U/L
Blood urea nitrogen	73.6	8–20 mg/dL
Creatinine	2.43	0.40–1.10 mg/dL
eGFR	20.3	>60.0 mL/min/L
Serum Na	130	135–150 mEq/L
Serum K	6.0	3.5–5.3 mEq/L
Serum Cl	99	98–110 mEq/L
Serum Ca	9.4	8.8–10.2 mg/dL
CK	85	56–244 U/L
CK-MB	3	<5 mg/mL
CRP	0.80	<0.30 mg/dL
Serum glucose	159	70–110 mg/dL
TSH	3.00	0.35–4.94 μIU/mL
Free T4	0.9	0.70–1.48 ng/dL
Troponin I	0.111	0.000–0.029 ng/mL
Brain natriuretic hormone	1360.5	<18.4
Lupus anticoagulant (Silica clotting time ratio)	0.51	<1.16
Anti-cardiolipin antibody	<0.4	<12.3 U/mL
Urine test		
Leukocyte	Negative	Negative
Nitrite	Negative	Negative
Protein	2+	Negative
Glucose	4+	Negative
Urobilinogen	Negative	Negative
Bilirubin	Negative	Negative
Ketone	Negative	Negative
Blood	3+	Negative
pH	5.5	5.0–7.5
Pleural effusion		
pH	7.326	
Total protein	1.3 g/dL	
Lactate dehydrogenase	87 U/L	
Glucose	125 mg/dL	
Adenosine deaminase	13.7 U/L	

**Figure 1:**
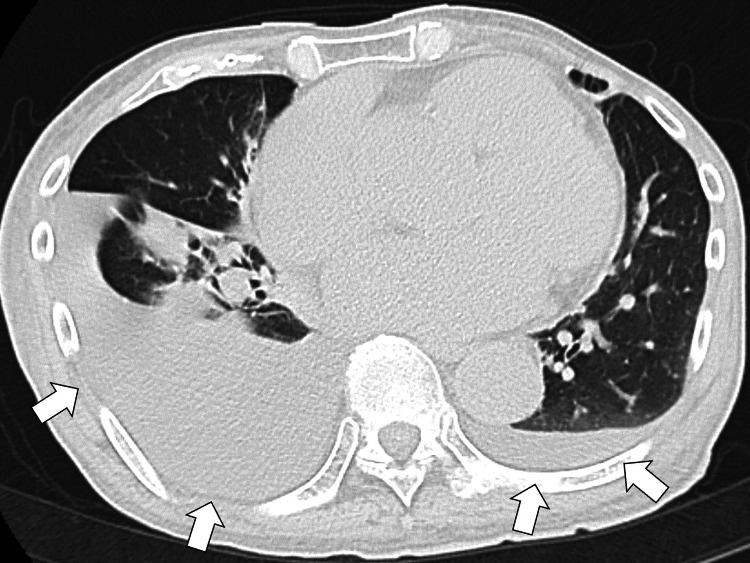
Thoracoabdominal computed tomography shows bilateral pleural effusions with no pleural or pericardial thickening (white arrows).

Chest radiography revealed a cardiothoracic ratio of 57%, signs of pulmonary congestion, and decreased right lung permeability. Transthoracic echocardiography revealed a left ventricular ejection fraction of approximately 10%, diffuse hypokinesis, mild mitral regurgitation, and tricuspid regurgitation, but no pericardial effusion, aortic valve stenosis, or D-shape. The diameter of the inferior vena cava was enlarged, but no respiratory changes were observed. Thoracoabdominal computed tomography (CT) revealed bilateral pleural effusions and a small amount of ascites but no pleural or pericardial thickening (Figure 1).

The ascending aorta measured 55 mm in short diameter and was enlarged. There was no thickening or enlargement of the gallbladder wall or increase in the peri-gallbladder fatty tissue density, despite the presence of stones in the gallbladder.

These findings indicate congestive heart failure was diagnosed because of cardiomegaly, jugular venous distention, and pleural effusion. Vital signs were stable, but the peripheral circulation was cold, and the serum lactate level was elevated. The patient was diagnosed with peripheral circulatory failure and cardiogenic shock due to a marked decrease in cardiac output. Elevated hepatic enzyme levels and worsening renal dysfunction were attributed to congestion of the right heart system and circulatory disturbances. Dobutamine was used to increase cardiac output, furosemide was used for fluid control, and nicardipine was initiated because of the continued elevation of blood pressure. The patient also had gallstones and right quadriceps pain, and the possibility of sepsis due to cholecystitis could not be ruled out; therefore, tazobactam/piperacillin was started. Echocardiography and serum lactate measurements were performed to follow up on the left ventricular ejection fraction and peripheral circulatory failure, and the dobutamine dose was tapered off the following day. Antimicrobial therapy was terminated on the seventh day after blood cultures tested negative.

On the eighth day of hospitalization, the prednisolone dose was reduced to 7.5 mg; the following day, the patient's food intake decreased again, and his inflammatory response increased. We considered the possibility that a steroid-responsive immune complex vasculitis state was the cause of his generalized symptoms and reviewed the previous laboratory findings. Blood tests before admission revealed an 80-fold increase in antinuclear antibodies (nucleolar), leukopenia <4,000/μL, thrombocytopenia <100,000/μL, urine protein >0.5 g/day, and a decrease in both C3 and C4, which scored 15 points on the European League Against Rheumatism/American College of Rheumatology (EULAR/ACR) classification criteria 2019 [10]. Because the patient was over 50 years old and diagnosed with late-onset SLE, on the 16th day of hospitalization, the prednisolone dose was increased to 30 mg, the patient's urinary protein and C-reactive protein (CRP) levels decreased, and the course of SLE treatment was uneventful. The patient underwent nutritional therapy for decreased food intake and rehabilitation to maintain and increase his activity levels. On the 31st day, he was discharged to a nursing home.

## Discussion

In the present case, we focused on a course that a single disease could not explain. Through repeated examinations and tests, we diagnosed late-onset SLE, a rare and difficult-to-diagnose disease. Considering the similarities in the clinical characteristics of older and younger patients, more attention should be paid to patients with late-onset SLE. An aging society requires a flexible and multifaceted perspective. This study focuses on the diagnostic difficulties and clinical lessons learned from the diagnosis of late-onset SLE.

Diagnosing late-onset SLE in the elderly is more complex than in younger patients because of the higher prevalence of comorbidities and the need for differentiation from other diseases. Because SLE is more commonly associated with young women, it can be challenging to consider it a potential differential diagnosis in older patients [11]. The time from onset to diagnosis has been reported to be twice as long in patients with late-onset SLE as in younger patients [12]. In the present case, purpura on the anterior aspect of the lower leg and painful aphthous ulcers in the oral cavity were observed as skin symptoms. However, malar rash, photosensitivity, or discoidal rashes were not observed. In general, purpura is nonspecific to SLE compared to malar rash and photosensitivity. Oral aphthous ulcers in SLE are rarely painful and generally considered painless [13]. In this case, the inflammatory response was decreased by prednisolone, and the aphthous ulcer appeared simultaneously, suggesting the possibility that the cause was not SLE but other diseases such as herpes simplex virus infection.

Late-onset SLE is associated with less significant organ damage, such as nephritis and neural lupus, and has a more gradual disease course than early-onset SLE. In contrast, late-onset SLE is associated with a significantly higher mortality rate, and death from cardiovascular diseases unrelated to SLE is more common [8,14,15]. Good disease activity does not guarantee a benign course in elderly patients with comorbidities. We suspected that pneumonia or heart failure alone could not explain this condition. Through repeated blood tests and physical examinations, we identified a difficult-to-diagnose disease: late-onset SLE [16]. In addition to disease knowledge, collaboration with other professionals, including general physicians, nurses, and laboratory technicians, is essential because of the need for frequent blood tests and the importance of observing detailed physical findings [17,18].

## Conclusions

We reported a case of late-onset SLE that began with systemic symptoms and progressed to multiple organ failure. Early diagnosis and therapeutic intervention are important because the diagnosis of late-onset SLE is difficult and, in some cases, can be fatal owing to multiple organ failure. When unexplained systemic inflammation is encountered, considering SLE as a potential differential diagnosis, regardless of the patient’s age, can lead to improved diagnostic outcomes.
